# Under representation of people with epilepsy and intellectual disability in research

**DOI:** 10.1371/journal.pone.0198261

**Published:** 2018-06-21

**Authors:** Rohit Shankar, Charles Rowe, Alje Van Hoorn, William Henley, Richard Laugharne, David Cox, Raj Pande, Ashok Roy, Josemir W. Sander

**Affiliations:** 1 Cornwall Partnership NHS Foundation Trust, Bodmin, United Kingdom; 2 University of Exeter Medical School, Exeter, United Kingdom; 3 Coventry and Warwickshire NHS Trust, Solihul, United Kingdom; 4 Chair of the Faculty of Psychiatry of Intellectual Disability - Royal College of Psychiatrists, London, United Kingdom; 5 NIHR University College London Hospitals Biomedical Research Centre, UCL Institute of Neurology, Queen Square, London United Kingdom; 6 Chalfont Centre for Epilepsy, Chalfont St Peter, Buckinghamshire, United Kingdom; 7 Stichting Epilepsie Instellingen Nederland (SEIN), Heemstede, Netherlands; Nathan S Kline Institute, UNITED STATES

## Abstract

**Purpose:**

One quarter of people with epilepsy have an intellectual disability (ID) and one fifth of people with an ID have epilepsy. Both conditions are associated with higher levels of morbidity, stigma and premature mortality. There have been calls for action to promote more research in this group. We examined if this group are represented adequately in current research.

**Methods:**

**T**he proportion of research output in epilepsy conferences and publications relevant to ID and the proportion in ID conferences and publications on epilepsy for 2015–2016 were identified. As the percentage of children in the population with epilepsy is 17%, research output of this group was compared with the ID group. Recognised material was classified based on whether it applied to general epilepsy/ID research, children with epilepsy or people with epilepsy and ID. Data was analysed to determine the proportion of presented research specifically identifying people with epilepsy and ID.

**Results:**

Fewer than 2% of presentations at epilepsy conferences specifically related to the ID and epilepsy group compared to 15% relating to children with epilepsy. Similarly only 1.4% of the research presented at major ID conferences related to those with people with epilepsy and ID. About 5% of published research in the field of epilepsy related to those with ID as compared with 24% for children with epilepsy. Twelve percent of published research in ID specifically identified epilepsy.

**Conclusion:**

Publications and conference presentations, on the population with epilepsy and comorbid ID is under-represented. Increased research in this area might assist in improving the quality of care for this relatively neglected group.

## Introduction

Approximately 20% of those with Intellectual Disability (ID) have comorbid epilepsy [1, 2]. The incidence of epilepsy rises with increasing severity of ID [1, 2]. Estimates also consistently find that 25% of all people with epilepsy have ID [3, 4, 5, 6]. Epilepsy management represents an important healthcare need for many of those with ID. For over a decade now the leading UK charity MENCAP has highlighted the continued inequality in healthcare provision to those with ID [7]. The inadequate treatment of people with epilepsy and ID may lead to premature mortality. This has been highlighted in a recent call for action by international epilepsy experts who have discussed failures in recognising and preventing epilepsy related mortality in this population [8]. Lack of research on preventive interventions was one area where action was called for, as generating a suitable evidence base may assist in addressing the inequality of healthcare provision. To date, little effort seems to have been put into identifying or quantifying this inequality.

People with comorbid epilepsy and ID are potentially at risk of poor treatment for both of their conditions. They carry the complexities of having both diagnoses and deserve special attention for a variety of reasons. People with ID have significant barriers to accessing healthcare. Communication and cognitive difficulties mean they often struggle to advocate satisfactorily for themselves. Complex issues about consent to treatment and mental capacity complicate care. This is compounded by a lack of understanding or experience by healthcare professionals and social stigma which still persists. There is a dearth of quality research specifically on this group and data from drug trials is scarce [1, 3]. Clinical trials of anti-epileptic drugs (AED) for example will often apply the presence of ID as an exclusion criterion [6]. Factors such as chronicity, seizure type and prognosis are often worse in comparison to the general epilepsy population [1, 3, 6]. This is compounded by the increased rate of treatment resistance in this population. Consequently, rates of multiple AED use are higher, with associated additional risks and treatment costs [6]. Seizures are the most common reason for avoidable emergency hospital admission in the ID population [9]. The incidence of sudden unexplained death in epilepsy (SUDEP) is also higher in this group [6].

Approximately 17% of the people with epilepsy are children [10, 11]. Both the groups of patients in general with intellectual disability and the group of children with epilepsy have important differences from the general epilepsy population and thus merit separate attention from the epilepsy research world. While not comparable as special poulations to each other in any clinical form it was felt it would be an useful and interesting exercise to see if research estimates match their individual prevalence rates respectively.

## Aim

We aimed to examine the following research questions:

1. What proportion of current research in people with epilepsy identifies comorbid ID
2. How does the amount of research undertaken compare with a population of children with epilepsy in relation to the prevalence of both populations.
3. What proportion of research in ID identifies people with comorbid epilepsy?

## Methods

We examined all major US, UK, and European conferences for either ID or epilepsy that took place in 2015 and 2016. Printed or digital conference programmes were obtained for analysis (S1 Appendix).

The conference programmes were analysed independently by two researchers. Epilepsy conference material was categorised as general epilepsy, People with epilepsy and ID or children with epilepsy. This included all seminars, workshops, oral and poster presentations. The categorisation was checked for agreement between researchers. Where research was difficult to categorise due to ambiguity or disagreement it was grouped as general epilepsy research.

Similarly for major ID conference programmes the presented material was classified as either general ID research or specific to epilepsy. Presentations on children with epilepsy and ID (if any) were included in the epilepsy group.

Due to its availability digitally an electronic search of abstracts was performed of the American Epilepsy Society’s (AES) annual meeting 2015. This method allowed using Boolean keyword searches to categorise the presented material at the conference (appendix 2).

We also estimated the proportion of current published literature in peer reviewed journals on epilepsy and ID. The years 2015–2016 were chosen.

A basic electronic database search was conducted across the following databases: BNI, CINAHL, EMBASE, Medline and Psychinfo. Using a series of Boolean keyword searches (S1 Appendix) the number of published articles with titles or abstracts relating to ID health related topics and epilepsy was obtained. A further search was performed of these to find the number pertaining to children with epilepsy and comorbid epilepsy and ID. It would not have been possible to categorise all the various epilepsy-linked ID syndromes using our search terms and this was consequently not undertaken.

Uncertainty in the estimated proportions is presented using 95% confidence intervals, calculated using Agresti and Coull’s method for interval estimation for a proportion. This method has been shown to provide better coverage probabilities than the standard Wald confidence interval, and is recommended for use when the sample size is more than 40 [12]. Differences in proportions between groups were identified by looking for non-overlap of confidence intervals and by conducting Fisher’s exact test.

## Results

There were only 2 instances of disagreement between the 2 reviewers. Both instances were added to the general epilepsy group. It is unlikely, given the small numbers of disagreements, that this would have had any major bias on the final outcomes.

### Epilepsy related conferences

Manual analysis of the three major epilepsy related conferences in Europe 2015–16 (Table 1) identified 1837 research items in the field of epilepsy of which 32 (1.7%; 95% CI: 1.2% to 2.5%) were ID related and 272 (14.8%; 95% CI: 13.3% to 16.5%) child related.

**Table 1:** Manual search of epilepsy conferences.

	Epilepsy Total	Epilepsy in children(excluding ID)	Epilepsy and ID (PWE-ID)
12^th^ European Congress on Epileptology 2016-Prague	959	130 (13.6%)	18 (1.9%)
The ILAE 31^st^ International Epilepsy Congress 2015-Istanbul	796	138 (17.3%)	11 (1.4%)
The 1^st^ Congress of the European Academy of Neurology 2015-Berlin	82	4 (4.9%)	3 (3.7%)
**TOTAL**	**1837**	**272 (14.8%)**	**32 (1.7%)**

When considering the 1^st^ Congress of the European Academy of Neurology 2015, of the 82 studies in the epilepsy section, three posters/presentations (3.7%; 95% CI: 0.9% to 10.8%) presented related to ID. The confidence interval for the PWE-ID percentage here has substantial overlap with the CI for the manual search above. Fisher’s exact test indicated that no statistical significance could be established (p = 0.17). There was no statistical difference between the amount of research on PWE—ID to that on children with epilepsy, with four posters/presentations (4.9%; 95% CI: 1.6% to 12.4%). Overall the epilepsy research material presented at this neurology conference was low with only 82 posters/presentations, roughly 10% of the total content of the conference, but the representation of people with ID was similar to the children with epilepsy group.

The ILAE 31^st^ International Epilepsy Congress 2015 had 138/796 posters/presentations (17.3%; 95% CI: 14.9% to 20.1%) of items on children with epilepsy. The proportion of research on epilepsy and ID was however small in comparison with only 11 posters/presentations of research (1.4%; 95% CI: 0.7% to 2.5%).

At the 12^th^ European Congress on Epileptology 2016 research on epilepsy and ID was 1.9% (18 pieces; 95% CI: 1.2% to 3.0%) compared to 13.6% (130 posters/presentations; 95% CI: 11.5% to 15.9%) on children with epilepsy.

An electronic abstract search of the 2015 annual meeting of American Epilepsy Society (Table 2) only identified 0.5% (95% CI: 0.1% to 1.4%) of the abstracts relating to epilepsy and ID. Children with epilepsy featured in 11.0% (95% CI: 8.8% to 13.7%) of the research presented.

**Table 2:** American epilepsy society conference electronic search.

	Epilepsy Total	Epilepsy and children(excluding ID)	Epilepsy and ID (PWE-ID)
Annual meeting of the American Epilepsy Society Philadelphia2015	645	71 (11.0%)	3(0.5%)

### ID related conferences

Two conferences were deemed suitable and equitable to the international stature of the epilepsy conferences and analysed (Table 3). Only 1.4% (95% CI: 0.6% to 3.1%) of the total research material pertained to epilepsy. The remaining topics were other general ID related health research.

**Table 3:** Manual search of ID conferences.

	General ID health related research	People with epilepsy and ID (PWE-ID)
IASSID 16^th^ World Congress 2016—Melbourne	370	3 (0.8%)
RCPsych Faculty of Psychiatry of Intellectual Disability Conference 2015—Newcastle	60	3 (5%)
**TOTAL**	**430**	**6 (1.4%)**

The UK based Royal College of Psychiatrists Annual ID conference had better representation of epilepsy with 3/60 posters/presentations (5%; 95% CI: 1.2% to 14.4%) of research material compared with only 3/370 (0.8%; 95% CI: 0.2% to 2.5%) of material presented at the International Association for Scientific Study of Intellectual and Developmental Studies (IASSID) 16^th^ World Congress (Fisher’s exact test p = 0.04).

### Epilepsy related database search

Database searches revealed that overall only 2,482 articles (4.9%; 95% CI: 4.7% to 5.1%) published 2015–2016 on epilepsy and ID compared to 12,140 articles (24%; 95% CI: 23.6% to 24.3%) relating to children with epilepsy (Table 4). The proportion of articles relating to epilepsy and ID across the more medical databases Psychinfo, Medline and Embase was 4.9% (95% CI: 4.8% to 5.1%). The nursing and allied health related databases BNI and CINAHL had a lower proportion of research on the target group with 3.8% (95% CI: 1.4% to 8.9%) and 3.2% (95% CI: 2.4% to 4.3%) respectively. The BNI database had the greatest proportion of articles published relating to children with epilepsy (37.1%; 95% CI: 29.4% to 45.6%). Across the other databases between 20–25% of published material on children with epilepsy.

**Table 4:** Epilepsy related database search.

	Epilepsy in Total	Epilepsy and children	People with epilepsy and ID (PWE-ID)
BNI	132	49 (37.1%)	5 (3.8%)
CINAHL	1349	332 (24.6%)	43 (3.2%)
EMBASE	27993	7070 (25.3%)	1390 (5%)
Medline	16210	3550 (21.9%)	797 (4.9%)
Psychinfo	4996	1139 (22.8%)	247 (4.9%)
**TOTAL**	**50680**	**12140 (24%)**	**2482 (4.9%)**

### ID related research database search

Database searches revealed that of the total ID health related articles (20,200) published during 2015–2016, 2,482 (12.3%; 95% CI: 11.8% to 12.7%) were on epilepsy (Table 5). Embase had the highest proportion of ID research identified on epilepsy (16.6%; 95% CI: 15.8% to 17.4%), the more nursing and allied health related databases BNI and CINALH having much smaller proportions of 1.4% (95% CI: 0.5% to 3.5%) and 3.6% (95% CI: 2.7% to 4.9%) respectively.

**Table 5:** ID related research database search.

	ID health related material	People with epilepsy and ID (PWE-ID)
BNI	353	5 (1.4%)
CINAHL	1181	43 (3.6%)
EMBASE	8388	1390 (16.6%)
MEDLINE	6862	797 (13.1%)
Psychinfo	3416	247 (11.6%)
**TOTAL**	**20200**	**2482 (12.3%)**

## Discussion

Our results build a picture of significant under-representation of people with epilepsy and ID in recent research. All of the major epilepsy conferences analysed showed a greater and more equitable proportion of research in children with epilepsy compared with people with ID.

There was only one major international ID conference in the years 2015–2016. Disappointingly of the 370 posters/presentations of general ID research presented at this conference only 3 posters/presentations were relevant to epilepsy which was 0.8% of all the conference material. The UK Royal College of Psychiatrists ID conference 2015 included 5% of topics relating to epilepsy, the highest of any of the conferences. It could be argued, however, given the significance of epilepsy in the ID population, where epilepsy is the most common chronic cause of premature mortality in this population, this is still inadequate.

It is worth mentioning that the results in the European conferences and RCPsych ID conference will have been influenced and biased by the work of the co-authors of the study (five for epilepsy and four for ID conferences respectively). The bias would have the risk of reducing the actual difference. Nevertheless even with this bias there are differences which cannot be neglected.

Database searches highlighted a similar trend of under-representation of the ID population with epilepsy, although the share of research papers was higher. The proportion of papers identified as looking into ID specific issues in epilepsy related journals was 4.9%. In 12.3% of ID related journals epilepsy was a significant subject.

We found 24% of the total published epilepsy research was on children. This is more than population estimates that roughly 17% of population with epilepsy are children.

There is no stigma attached to research in children with medical conditions in general; it could be argued that funding for medical research in children would, by many, be considered as being more attractive than funding than for medical research in adults. The same argument is not true for adults with intellectual disability. This group is likely to be stigmatised. In both cases, the epilepsy itself may be associated with some stigma.

## Limitations

Manual classification of conference material into different categories is open to error due to the subjectivity of this system. There is also a chance small amounts of material could have been misclassified or counted inaccurately especially as ID researchers doing the searches could be more sympathetic to people with ID. To minimise these limitations categorization was done independently by the two researchers.

Database searches have the advantage of allowing the collection of a large data set, but the presence of a keyword when searching abstracts does not always mean that it is the main, or even an important subject of the paper. The searches were designed to show the overall trend. This limitation might influences proportions in the different searches undertaken and possibly account for the increase in proportion of research in database searches compared to conferences. The likelihood of the limitations significantly biasing or influencing the discussion and conclusions of the study is low.

The use of the paediatric group is to give an example of how proportionate research is in a well known special population which has fewer issues of stigma and funding than the mainstream. It is to help provide a comparator to identify the levels of research the ID epilepsy population could aspire to. It also serves as a useful benchmark to highlight how ID is possibly at variance from other special populations.

## Conclusion

People with ID and epilepsy appear to be under-represented in both epilepsy and ID research fields respectively. Our analysis suggests that publications and conference presentations on people with ID and epilepsy are under-represented.

In view of the clear evidence showing that people with both ID and epilepsy tend to have poor health and social outcomes, placing increased demands on health and care costs, there is a strong case for promotion of research in this vulnerable group. The aim of such a research should be to promote improved quality of care in the future.

### APPENDIX 2: Boolean search algorithms for database search

**Search protocol for AES 2015**
- Total material pertaining to general epilepsy research = Total number of items presented (all search terms)
- Search terms for PWE-ID = ((learning disab* OR intellectual disab* OR mental retard* OR developmental disab* OR cognitive disab* OR low IQ) AND (epilep* OR seiz* OR convuls*) NOT febrile).ti,ab [DT 2015–2016]
- Search terms for children with epilepsy

**Search protocol for method 2**
- Total published articles pertaining to general epilepsy topics = (epilep* OR seiz* OR convuls*).ti,ab [DT 2015–2016]
- Total published articles pertaining to ID health related research = (learning disab* OR intellectual disab* OR mental retard* OR developmental disab* OR cognitive disab* OR low IQ).ti,ab [DT 2015–2016]
- Search terms for children with epilepsy = ((epilep* OR seiz* OR convuls*) AND (child* OR adolesc* OR paediat* pediat* OR neonata OR infa* OR juve*) NOT febrile).ti,ab [DT 2015–2016]
- Search terms for PWE-ID = ((learning disab* OR intellectual disab* OR mental retard* OR developmental disab* OR cognitive disab* OR low IQ) AND (epilep* OR seiz* OR convuls*) NOT febrile).ti,ab [DT 2015–2016]

## Supporting information

S1 Appendix (DOCX)
