# Supplementary figures and images for: Under representation of people with epilepsy and intellectual disability in research
Source: PLoS One. 2018 Jun 21;13(6):e0198261. doi: 10.1371/journal.pone.0198261 (PMC6013187; doi:10.1371/journal.pone.0198261)

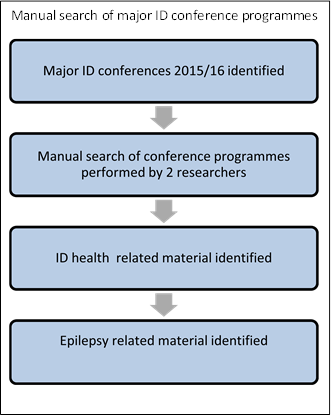

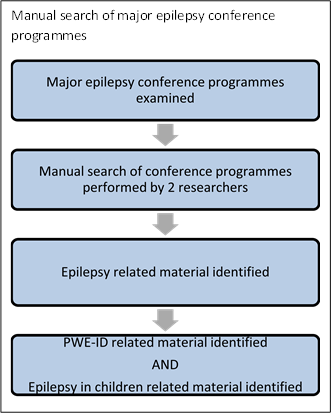

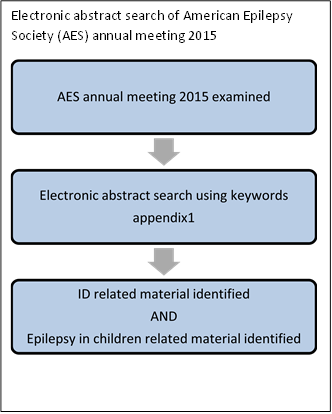

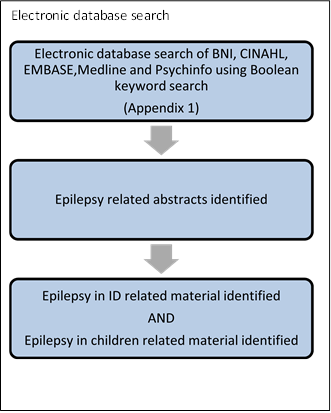


S1 Appendix

Supplement: S1 Appendix — (DOCX) [file pone.0198261.s001.docx]
